# Study protocol of an exercise and nutrition intervention for ovarian cancer patients during and after first-line chemotherapy (BENITA) - a randomized controlled trial

**DOI:** 10.1186/s12885-024-13102-y

**Published:** 2024-11-11

**Authors:** Tabea Maurer, M. H. Belau, B-C. Zyriax, G. Welsch, B. Jagemann, J. Chang-Claude, A. Daubmann, A. Buchholz, K. Glismann, A. Moeller, J. Sehouli, H. Woopen, P. Wimberger, P. Harter, S. Kaiser, N. Maass, M. Kiechle, T. Engler, B. Schmalfeldt, H. Schulz

**Affiliations:** 1 Center for Psychosocial Medicine, Department of Medical Psychology, University Medical Center Hamburg-Eppendorf, Hamburg, Germany; 2 Institute of Medical Biometry and Epidemiology, University Medical Center Hamburg-Eppendorf, Hamburg, Germany; 3 Midwifery Science-Health Care Research and Prevention, Research Group Preventive Medicine and Nutrition, Institute for Health Service Research in Dermatology and Nursing (IVDP), University Medical Center Hamburg-Eppendorf, Hamburg, Germany; 4 Orthopedic Sports Medicine, Department of Trauma and Orthopedic Surgery, UKE Athleticum, University Medical Center Hamburg-Eppendorf, Hamburg, Germany; 5 Division of Cancer Epidemiology, German Cancer Research Center (DKFZ)University Medical Centre Hamburg-Eppendorf, University Cancer Centre Hamburg (UCCH), Heidelberg, Germany; 6 Department of Gynecology with Center for Oncological Surgery, Charite Comprehensive Cancer Center, Charité – Universitätsmedizin Berlin, Corporate Member of Freie Universität Berlin and Humboldt- Universität zu Berlin, Berlin, Germany; 7 National Center for Tumor Diseases Dresden, Department of Gynecology and Obstetrics, University Hospital Dresden, TU Dresden, Dresden, Germany; 8 Department of Gynecology & Gynecologic Oncology, Ev. Kliniken Essen-Mitte, Essen, Germany; 9 Department of Gynecology and Obstetrics, University Hospital of Schleswig-Holstein, Campus Kiel, Kiel, Germany; 10 Department of Gynecology and Obstetrics, University Hospital Rechts der Isar, Technical University Munich (TU), Munich, Germany; 11 Department of Women’s Health, Tübingen University Hospital, Tübingen, Germany; 12 Department of Gynecology, University Medical Center Hamburg-Eppendorf, Hamburg, Germany

**Keywords:** Ovarian cancer, Randomized controlled trial, Exercise, Nutrition, Malnutrition, Muscle wasting

## Abstract

**Background:**

In ovarian cancer frequently reported side effects are muscle wasting and malnutrition, leading to frailty, decreased health-related quality of life (HRQoL), and cancer-related fatigue (CRF). Both often begin during first-line chemotherapy and develop progressively into a refractory state, if left untreated.

**Method:**

Primary objective is to evaluate effectiveness of a newly developed app-based exercise and nutrition program under non-standardized conditions of clinical routine. We hypothize that patients who receive an individually tailored exercise and nutrition program for six months will have improved physical performance compared to patients who receive usual care. This is a multicenter randomized controlled open-label trial comparing an intervention group receiving a six-month exercise and nutrition intervention and a control group receiving usual care. Primary endpoint is the change in 6-Minute Walk Test (6MWT) from baseline to T2 (26 weeks after baseline) as a measure of physical functioning. Secondary endpoints include patients’ utilization and adherence to the nutrition program (MEDAS), their malnutrition risk (NRS2002), as well as patients’ HRQoL (see Table 1). Using the two-sample t-test with a two-sided type I error of 5% and 80% power, a medium effect size of Cohen’s d = 0.50 can be demonstrated with a minimum of 128 participants (64 per group). With a conservatively estimated dropout rate of 30%, 182 patients will be recruited. Patients who are included must be over 18 years of age, be diagnosed with ovarian cancer, cancer of fallopian tubes, or peritoneal cancer, FIGO stages II-IV, receive surgery and chemotherapy (adjuvant or neoadjuvant). Exclusion criteria are an ECOG status greater than 2, inadequate proficiency in German, or physical or mental impairments hindering the implementation of the program or execution of study procedures.

**Discussion:**

In case of success, the project contributes in the long term to (i) improving medical care (diagnosis, psychoeducation, patient orientation, and empowerment), (ii) reducing the burden of disease and promoting physical autonomy for patients, and (iii) being incorporated into relevant guidelines.

**Trial registration:**

The study was registered at ClinicalTrials.gov (NCT06250686).

## Background

Ovarian cancer is the second most common gynecological cancer with the highest mortality rate in women [1]. It primarily affects older women and is often diagnosed at a late stage due to its inconspicuous and non-specific initial symptoms. Patients typically suffer from peritoneal carcinosis, ascites, and digestive tract involvement. Treatment involves extensive surgery to remove the tumor, followed by aggressive chemotherapy and maintenance therapy. A common side effect of the tumor and its treatment is called tumor cachexia. It is characterized by muscle wasting and malnutrition, which, if left untreated, can develop into an irreversible condition. Ovarian cancer patients exhibit a 19-fold increased risk of malnutrition compared to women without malignant tumors and the majority of women diagnosed with ovarian cancer lead sedentary lifestyles. More than half of these patients even report completely inactive behavior [2]. Studies on other cancer entities, such as breast cancer, have shown that an exercise and nutrition program can have a positive impact on physical performance, quality of life, and even patient survival [3]. Initial evidence also suggests that healthy nutrition following diagnosis could improve the survival outcomes of ovarian cancer patients [4]. Adherence to recommended dietary guidelines before and after cancer diagnosis has been shown to significantly improve quality of life [5] and reduce cancer mortality [6]. Limited available observational studies have shown that patients with higher physical activity after diagnosis exhibit significantly better quality of life [7]. Combined nutrition and exercise interventions may be most effective in addressing muscle wasting and malnutrition in advanced-stage cancer patients. One trial evaluating the role of improving peri-operative management including exercises and nutrition interventions has just closed recruitment [8]. However, there is a scarcity of randomized controlled trials (RCTs) evaluating the benefits of such bimodal interventions, especially for patients with ovarian cancer who represent a distinct patient group characterized by advanced age and the severity of the disease. Besides our feasibility study [9], there are currently six smaller studies demonstrating the feasibility of exercise and/or nutrition interventions in ovarian cancer patients during and after chemotherapy [10–16]. Furthermore, there are three larger randomized studies: The first study revealed that an exercise intervention improved the physical quality of life in ovarian cancer patients. The second study, involving a combined exercise and nutrition program, is still ongoing [17]. However, both studies investigate the effects of an intervention post-treatment, while research on cancer cachexia suggests that programs should intervene during treatment to prevent the development of a refractory condition. The third study is not published yet [18] but stated in their study protocol an intervention period of about 21 weeks which is a much shorter time-line compared to our study and therefore may not lead to the expected effects on physical performance.

Exercise and nutrition recommendations after ovarian cancer diagnosis are currently based solely on expert consensus, even in the current version of the German guideline for ovarian cancer (2021). Even though exercise and physical activity counseling to reduce fatigue during chemotherapy is recommended, there are no specific details about training programs. For general follow-up care, the German guideline “Supportive Therapy for Oncology Patients” is referenced, with likewise unspecific general recommendations regarding sports and exercise. Furthermore, for a significant proportion of patients, undergoing oncological rehabilitation is not feasible. Although the guidelines prescribe a 15–24 months maintenance therapy following primary treatment, many patients do not feel able to undergo further inpatient or outpatient treatment, often in a distant location [9]. Currently, there is no individually tailored program available that begins during active primary therapy and is fully integrated into clinical practice. There is also no concept for implementing these programs into clinical routine or evaluating them in clinical practice.

We hypothesise that patients who receive an individually tailored exercise and nutrition program adapted to their needs and capabilities for 6 months will have improved physical performance compared to ovarian cancer patients who receive standard care.

## Methods

### Trial design

A randomized controlled open-label trial conducted at seven study sites in Germany (Hamburg, Berlin, Essen, Dresden, Munich, Tubingen, Kiel) with assessments at baseline (T0, before randomization), 19 weeks (T1) and 26 weeks (T2) after T0. The total study duration is 28 months. It starts with 16 months of patient recruitment. After 22 months, all enrolled patients will have completed the trial (6-months period for the intervention), and at the end of 28 months, statistical analysis and publication of results will be concluded.

**Fig. 1:**
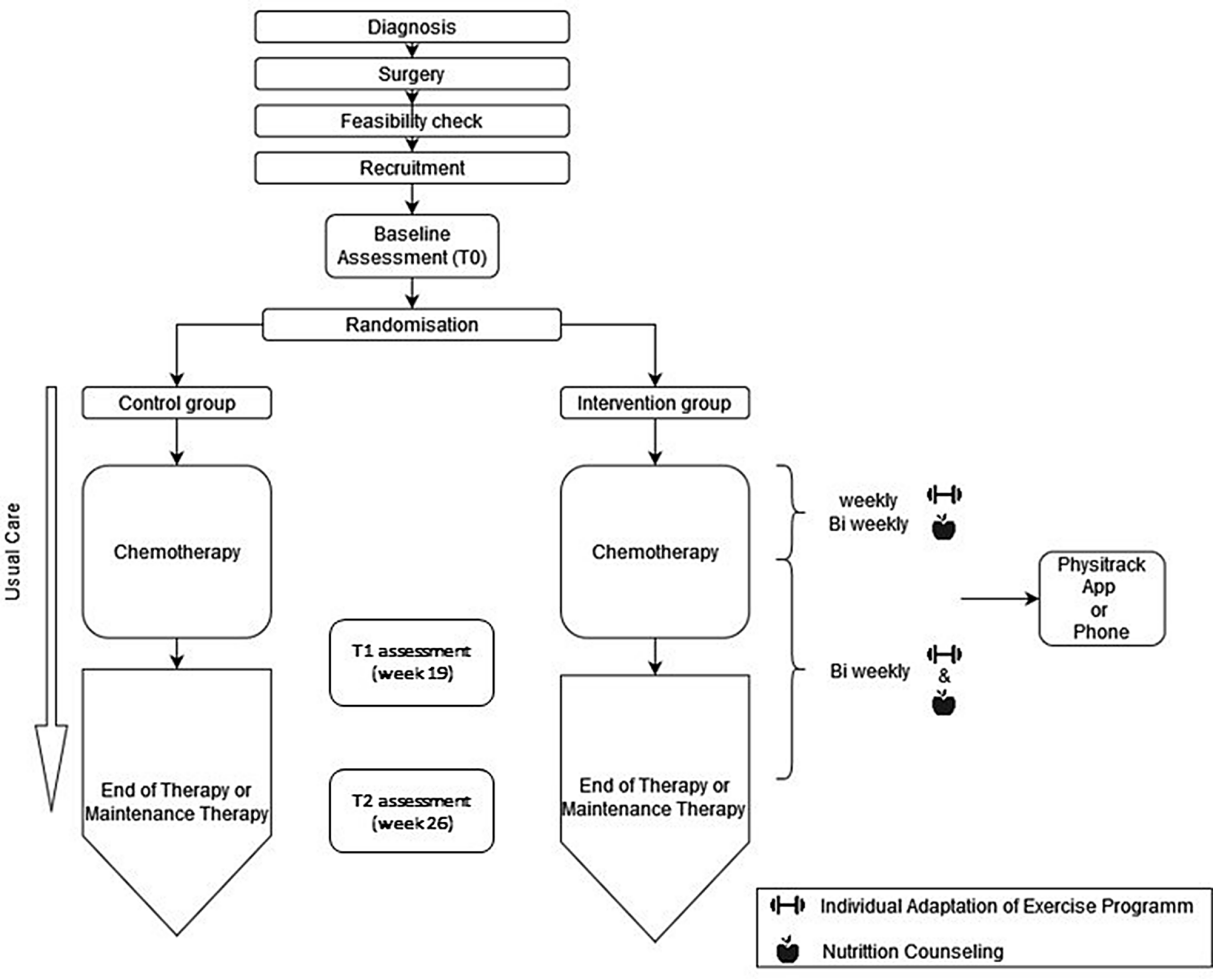
BENITA Study Design for patients receiving adjuvant chemotherapy: Patients of the intervention group receiving adjuvant chemotherapy start the exercise and nutrition program at T0. It continues for 26 weeks. Patients of the control group receive usual care

**Fig. 2:**
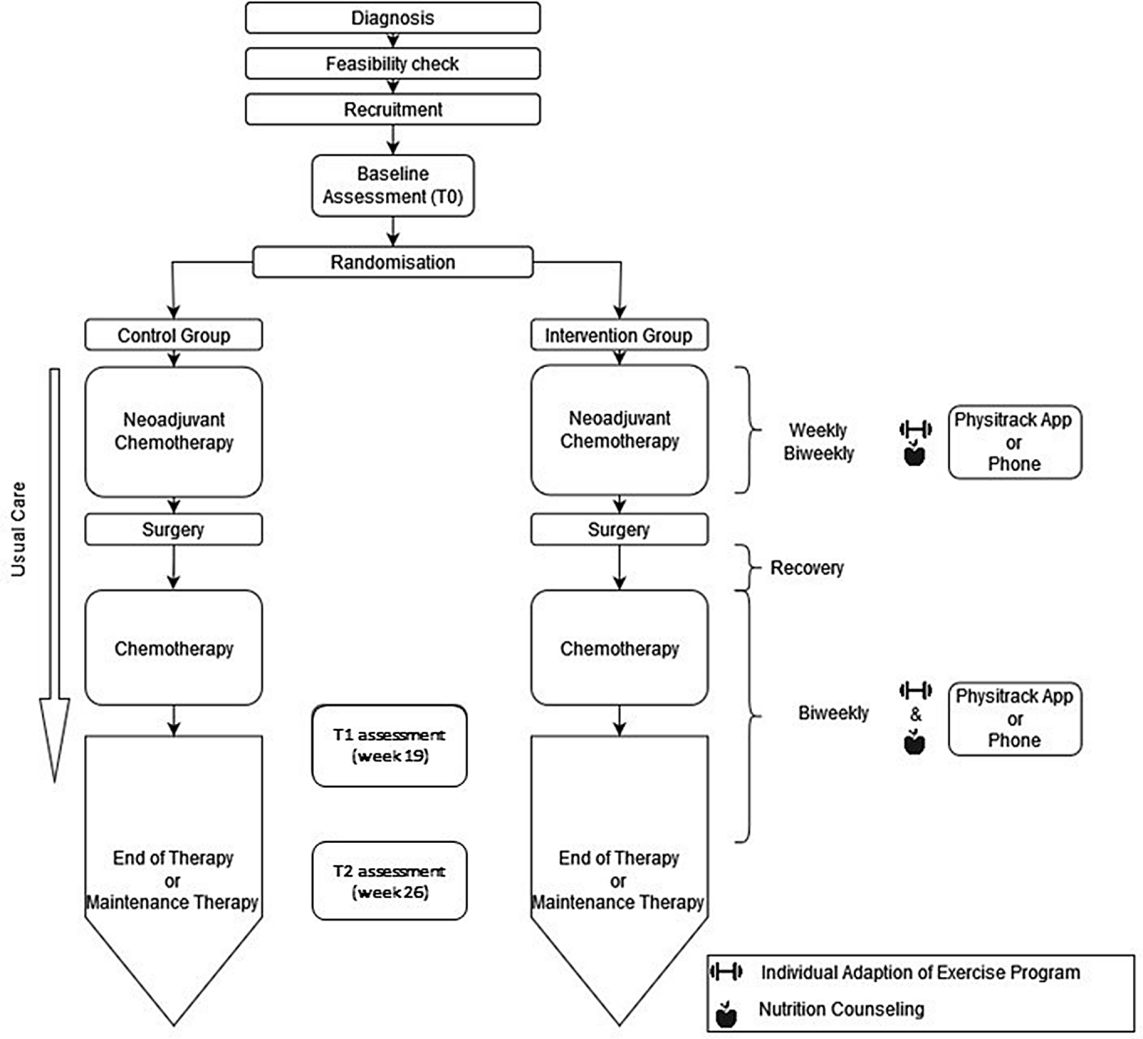
BENITA Study Design for patients receiving neoadjuvant chemotherapy: Patients of the intervention group receiving neoadjuvant chemotherapy start the exercise and nutrition intervention at T0. The intervention is paused during surgery and recovery. Patients of the control group receive usual care

Figures 1 and 2 depict the study design for patients receiving adjuvant and neoadjuvant chemotherapy, respectively. During counseling, patients randomized to the intervention group receive an app-based (Physitrack^®^, Bastion House, 6th Floor, 140 London Wall, London EC2Y 5DN, United Kingdom) personalized exercise and nutrition program tailored to the various phases of their treatment and recovery, as well as their individual needs. The basis for this program is the concept of exercise and nutrition applied during the pilot phase [9], which was optimized through semi-structured interviews with all stakeholders in a pre-study conducted before the main trial. The program consists of two stages. In stage I (Week 1 to 18), starting with the initiation of chemotherapy, a daily training regimen lasting approximately 15 to 30 min has been developed. This includes endurance, resistance, mobility, balance exercises with gradual intensity progression. Nerve gliding exercises are included if symptoms of polyneuropathy occur due to chemotherapy. Abdominal muscle exercises are to be introduced only after complete recovery from surgery. Every 2nd week the training program is adapted to the patient’s needs after the weekly or bi-weekly patient consultations via video call or telephone. The nutritional counseling in stage I aims to assess and potentially reduce the individual risk of malnutrition (protein-energy malnutrition) according to guidelines. Based on individual information gathered with validated instruments (Nutritional Risk Screening (NRS)-2002, 3-day dietary records, MEDAS questionnaire), nutrition plans with recipes are provided. Attention is given to chemotherapy side effects that may adversely affect patients’ eating habits. Nutritional counseling aligns with the World Cancer Research Fund (WCRF)/American Institute for Cancer Research (AICR) recommendations for cancer survivors. In stage II (Week 19 to 25) after completing chemotherapy, the intensity and duration of daily training increases. Nutritional counseling focuses more on adherence to the Mediterranean Diet, which ensures the necessary nutrient supply during cancer therapy. Both, exercise and nutrition intervention programs are designed and supervised by certified experts.

### Participants

Included in this study are female patients aged 18 years and older with ovarian cancer, cancer of fallopian tubes, or peritoneal cancer at FIGO stages II-IV, who are undergoing surgery and chemotherapy. Both patient subgroups receiving adjuvant and neoadjuvant chemotherapy, but who have not yet started treatment, are eligible for inclusion. Exclusion criteria are patients with an Eastern Cooperative Oncology Group (ECOG) performance status greater than 2, as well as patients with insufficient German language skills. Patients with physical or mental impairments that would interfere with the implementation of the training programs or the execution of the study procedures are also excluded.

### Endpoints

The primary endpoint is physical performance measured by the change in the total distance (in meters) of the 6-minute walking test from T0 to T2. Table 1 shows all endpoints, data collection time points and procedures.

**Table 1:** Primary and secondary endpoints, data collection time points, and procedures

Dimension	Test	Endpoint	T0	T1 (week 19)	T2 (week 26)
**Primary Endpoint**					
Physical performance	6-minute walking test	Changes in total distance (m)	X		X
**Secondary Endpoints**					
Physical performance	6-minute walking test	Changes in total distance (m)	X	X	
	GPAQ	Physical activity in minutes per day, MET-minutes per week	X	X	X
Physical condition	BIA-Measurement	R, XC, P, FM, FFM, BCM, ECM	X	X	X
	Grip strength dynamometer	Grip strength (kg)	X	X	X
	EORTC-CIPN 20 (Item 9)	Polyneuropathy	X	X	X
Emotional well-being	PHQ-9	Depression	X	X	X
	GAD-2	Anxiety	X	X	X
Quality of Life	EORTC QLQ-C30	PF, RF, EF, CF, GHS, FA, NV, PA, DY, SL, SF, AP, CO, DI, FI	X	X	X
	EORTC OV-28	GI, PN, CH, HM, BI, AT, SF2	X	X	X
	EORTC QLQ-FA12	Fatigue	X	X	X
Utilization and adherence to the program	MEDAS	Adherence to the Mediterranean diet	X	X	X
Nutrition	NRS2002	Malnutrition risk	X	X	X
Social support	BS6	Concrete (tangible) support, Emotional-informative support	X	X	X
Patient Activation	PAM-13	knowledge, beliefs, confidence, and skills about managing one’s health	X	X	X
**Quality Control**					
Utilization and Adherence to the Program	MEDAS (recorded in both treatment groups)	Adherence to the Mediterranean Diet			
	Training documentation in Physitrack and/or questioning (only in the intervention group)	Adherence to the Exercise Program			

BIA-measurements

R: Fluid status; XC: Capacitive reactance; P: Phase angle; FM: Body fat; FFM: Fat-free mass; BCM: Body cell mass; ECM: Extracellular mass)

EORTC scales

PF: Physical functioning; SF: Social functioning; GHS: Global health status; DY: Dyspnea; SL: Insomnia; GI: Abdominal/gastrointestinal symptoms; PN: Peripheral neuropathy; CH: Other chemotherapy-related side effects; HM: Hormonal/menopausal symptoms; BI: Body image; AT: Attitude towards illness and treatment; SF2: Sexual functioning)

Others

BS6: Brief social support scale; CIPN: Chemotherapy-induced peripheral neuropathy; EORTC: European Organisation for Research and Treatment of Cancer; GAD-2: generalized anxiety disorder 2-item questionnaire; GPAQ: Global physical activity questionnaire; MEDAS: Mediterranean Diet Adherence Screener; MET: Metabolic Equivalent of Task; NRS2002: Nutrition risk score; PAM-13: Patient activation measure; QLQ-C30: Quality of Life Questionnaire-Core; QLQ-FA 12: Quality of Life Questionnaire-Fatigue; QLQ-OV28: Quality of Life Questionnaire Ovarian Cancer

### Sample size calculation

Existing studies in patients with advanced tumor that have primarily investigated the effectiveness of a pure exercise intervention demonstrated effect sizes ranging from Cohen’s d = 0.20 to d = 0.80 [10, 11]. Considering the combined exercise and nutrition program we assume a medium effect size (Cohen’s d = 0.50) which is considered realistic and simultaneously of sufficient clinical relevance. Using a two-sample t-test, a two-sided significance level of 5% and a power of 80%, a medium effect size of Cohen’s d = 0.50 can be demonstrated (PASS 16.0.3) when at least 128 participants (64 per group) are available for analysis. Based on the BENITA feasibility study and the number of patients treated at each center, we expect per year approximately 38 patients from Berlin, 22 each from Hamburg, Essen, and Tubingen, and 11 each from Kiel, Dresden, and Munich to meet the study inclusion criteria and be willing to participate. Over the planned 16-month recruitment period, this would result in a total of 182 patients. Assuming a conservative dropout rate of 30%, the calculated necessary number of participants would still be available.

### Randomization and blinding

Patients will be randomly assigned with a ratio of 1:1 to one of the two arms (intervention group and control group) using a block randomization with varying block lengths, stratified by center and type of chemotherapy (adjuvant and neoadjuvant). The randomization will be performed using the Randomizer tool (www.randomizer.at), whose compliance with Good Clinical Practice (GCP) has been confirmed by the Austrian Federal Office for Safety in Health Care. The randomization list is stored in the Randomizer, and access for authorized users is encrypted via the Randomizer website, with all transactions being logged in the Randomizer. Blinding of patients and physicians is not feasible due to the study design and intervention program.

### Statistical methods

The analysis of the primary endpoint will be conducted using a mixed linear regression model. The model includes the difference in the 6-minute walking test from baseline as endpoint and treatment group, time point (categorical), the interaction between treatment group and time point, baseline value of the 6-minute walking test, type of chemotherapy, and patient’s overall physical activity level before diagnosis (binary, measured with the GPAQ, cutoff at 150 min of moderate-intensity physical activity per week or equivalent) as fixed effects. Additionally, the study center and the patient (nested within the study center) are included as random effects. The estimation will be performed using restricted Maximum-Likelihood with an independent covariance structure for the centers and a correlation structure for the repeated measurements within a patient, reflecting the dependence of the two measurement time points (exchangeable). The primary hypothesis will be tested using the treatment contrast at time point T2 (using the Wald test). The primary analysis will be conducted according to the intention-to-treat (ITT) principle. Missing values in the endpoint will not be imputed, but the implicit missing-at-random (MAR) assumption of the mixed model will be utilized.

Evaluation of the secondary endpoint change in the total 6-minute walking test distance at T1 (as a difference from baseline), will be conducted within the primary model through the treatment contrast at T1. Analysis of secondary endpoints, including physical activity in minutes per day and MET-minutes per week, as well as various dimensions of quality of life, nutritional status, physical condition, mental health, social support, and active patient participation, will also be analyzed using mixed linear regression models analogously to the primary endpoint. Analyses will be conducted according to the ITT principle without prior multiple imputations of missing values. All analyses of secondary endpoints and subgroups (intake of probiotics and social status), along with corresponding p-values, are exploratory. No adjustment for multiple testing will be made.

## Discussion

Ovarian cancer predominantly affects older women and is often diagnosed at a late stage of disease. A common side effect of the tumor and its treatment is known as tumor cachexia, characterized by muscle wasting and malnutrition. If left untreated, it can progress to an irreversible state [19]. In a long-term study on the consequences of chemotherapy in ovarian cancer patients, 40% exhibited poor physical performance, and 60% experienced low energy levels. Poor physical performance is further associated with an increased risk of additional symptoms such as pain, depression, or fatigue, crucial factors affecting the patients’ quality of life. Therefore, sustaining or enhancing patients’ physical abilities and improving nutritional status is of utmost importance and could support survivors in leading autonomous lives, thereby boosting their overall quality of life. Improved physical performance could also enhance therapy tolerance, adherence, and potentially positively impact rehabilitation after treatment and overall survival as shown in other cancer sites [20]. However, recommendations for exercise and nutrition, even in the current S3 guideline on ovarian cancer (2021), are solely based on expert consensus, lacking sufficient details on intervention selection, design, and acceptance. An individually tailored program, starting during active primary therapy and fully integrated into clinical practice, is currently not available. An exercise and nutrition program, accessible to all patients during treatment and adaptable for continued use by all patients even after completing therapy, would thus represent a significant improvement in the current healthcare situation.

Our exercise and nutrition program has already undergone a successful pilot phase, during which it was assessed for safety and acceptability [9]. In preparation of our main trial we further conducted semi-structured interviews with all relevant stakeholders (patients, survivors, physicians, sports and nutrition scientists, health insurance representatives). The findings from these interviews were used to further optimize the program and to ensure it implementability into clinical routine. By involving multiple centers across diverse geographic regions in the main study the approach not only enhances the external validity of our findings but also allows for the identification of potential regional differences. In terms of outcome assessment, we employed a comprehensive approach that included both objective medical endpoints as well as patient-reported outcomes. Hence, offering valuable insights into the physiological effects of the intervention, as well as subjective experiences and patient perspectives. Furthermore, we chose a centralized intervention, which is applied solemnly by experts located at the Hamburg study site. By establishing a central coordinating center responsible for overseeing intervention protocols and training personnel, we ensure consistency and adherence to standardized procedures across all study sites. This centralized approach minimizes variability in intervention delivery and enhances the reliability of study results. Moreover, by having all interventions conducted by the same team, we mitigate potential biases related to differences in expertise or practice styles across sites. Despite the strengths of our study design and methodology, several limitations warrant consideration and discussion. While our study included a control group, both patient groups are treated following the individual care as usual protocols of their respective study center. Furthermore, patients are free to seek support outside the hospital. We cannot control for or verify that participants in either group did not receive additional support related to nutrition and physical activity. However, this study aims to prove that an individualized intervention combining exercise and nutrition has a positive effect on patient’s physical functioning beyond currently practiced usual care. We also acknowledge that we will not be able to separate the effect of the exercise program from the effect of the nutrition program on patients’ physical functioning. Instead, we will be able to demonstrate an overall effect. In case of success, the project contributes in the long term to (i) improving medical care (diagnosis, psychoeducation, patient orientation, and empowerment), (ii) reducing the burden of disease and promoting physical autonomy for patients, and (iii) being incorporated into relevant guidelines.

## Data Availability

No datasets were generated or analysed during the current study.

## Abbreviations

6MWT: 6-Minute Walk Test
AICR: American Institute for Cancer Research
BENITA: Bewegungs- und Ernährungsintervention bei Ovarialkarzinom
BS6: Brief Social Support Scale
CIPN: Chemotherapy-Induced Peripheral Neuropathy
CRF: Cancer-Related Fatigue
ECOG: Eastern Cooperative Oncology Group
EORTC: European Organisation for Research and Treatment of Cancer
FIGO: International Federation of Gynecology and Obstetrics
GAD-2: Generalized Anxiety Disorder 2-item questionnaire
GCP: Good Clinical Practice
GPAQ: Global Physical Activity Questionnaire
HRQoL: Health-Related Quality of Life
MEDAS: Mediterranean Diet Adherence Screener
MET: Metabolic Equivalent of Task
NRS2002: Nutrition Risk Screening 2002
PAM-13: Patient Activation Measure
RCTs: Randomized Controlled Trials
QLQ-C30: Quality of Life Questionnaire-Core
QLQ-FA 12: Quality of Life Questionnaire-Fatigue
QLQ-OV28: Quality of Life Questionnaire Ovarian Cancer
WCRF: World Cancer Research Fund
